# Interprofessional Collaboration in Building In Situ Simulations to Identify Threats to Patient Safety Before Transitioning to a New Healthcare Environment: Neonatal Intensive Care as an Example

**DOI:** 10.7759/cureus.81178

**Published:** 2025-03-25

**Authors:** Ahmed Moussa, Audrey Larone Juneau, Charles-Olivier Chiasson, Laura Fazilleau, Justine Giroux, Marianne Lapointe, Émilie St-Pierre, Michael-Andrew Assaad, Jesse Bender, Beverley Robin

**Affiliations:** 1 Neonatology, University of Montreal, Montreal, CAN; 2 Centre de Pédagogie Appliquée aux Sciences de la Santé, University of Montreal, Montreal, CAN; 3 Neonatology, CHU Sainte-Justine, Montreal, CAN; 4 Pharmacy/Neonatology, CHU Sainte-Justine, Montreal, CAN; 5 Neonatology, CHU de Caen, Caen, FRA; 6 Neonatal-Perinatal Medicine, CHU Sainte-Justine, Montreal, CAN; 7 Neonatology, Virginia Tech Carilion School of Medicine and Research Institute, Roanoke, USA; 8 Neonatology, Rush University Children's Hospital, Chicago, USA

**Keywords:** in situ simulation, interprofessional education and collaboration, pediatrics & neonatology, simulation in medical education, single-patient rooms

## Abstract

Background and objective

While transitioning to a new healthcare environment (HCE) offers opportunities to enhance patient safety and outcomes, it can also introduce hidden risks. This study aimed to explore how interprofessional collaboration (IPC) and in situ simulations (ISS) can proactively identify and resolve these latent safety threats (LSTs) before transitioning to a new single-patient room neonatal ICU (NICU).

Methodology

We conducted a prospective, simulation-based intervention study involving healthcare professionals (HPs) and prior NICU parents. Three simulation activities were conducted to identify LSTs before the transition. The Canadian Interprofessional Competency Framework was employed to formulate realistic scenarios.

Results

A total of 108 HPs participated in six simulation sessions, identifying 89 LSTs across eight themes. The majority (76%) of these threats were resolved before the transition. Survey analysis revealed significant increases in systems readiness and staff preparedness post-simulations (p<0.001). Parental involvement significantly enhanced the focus on patient-centered care, leading to improvements in environmental design and communication systems.

Conclusions

The study demonstrates the efficacy of IPC and ISS in identifying and mitigating LSTs during HCE transitions, fostering a collaborative and safety-oriented culture. This approach prepares healthcare teams for new environments and emphasizes the value of incorporating family perspectives. Interprofessional ISS is a pivotal strategy to enhance patient safety and system readiness during transitions to new HCEs. The study also highlights the importance of IPC in conducting ISS before transitioning to a new HCE. Coordinating large-scale simulations is worth the time and cost investment necessary to identify LSTs, optimize systems readiness, and promote patient safety. We hope that the shared lessons can help future interprofessional teams in terms of plan testing and transitions to other HCEs.

## Introduction

Healthcare environments (HCEs) need to adapt in response to advancements in technology, changes in patient demographics, and the continuous quest for improved patient outcomes. The evolution of healthcare technologies, medical knowledge, and the growing need for human and material resources make it essential to optimize HCEs, to promote safe patient care. Integrating new and existing systems and workflows in a new environment can introduce latent safety threats (LSTs), which are unanticipated threats to patient safety [1]. These LSTs can materialize at any time and entail significant consequences for patients. Transitioning from traditional inpatient wards to single-patient rooms (SPRs) has been shown to be beneficial for adult and pediatric patients and their families: reduced risk of delirium in adult ICU and increased parental presence, lower maternal depression, and improved breastfeeding rates in the neonatal ICU (NICU) [2-6].

In situ simulations (ISS), conducted in the actual clinical environment, have been used to identify and mitigate LSTs before transitioning patients to new adult, pediatric, and neonatal HCEs [1,7,8]. ISS also facilitates the immersion of healthcare teams in the new HCE, which has been shown to improve their preparedness and readiness for the transition [9]. Preparation and implementation of ISS should be conducted through interprofessional collaboration (IPC) [10]. An IPC approach integrates diverse points of view, promotes a holistic assessment, and encourages effective communication between healthcare professionals (HPs). We conducted interprofessional ISS before transitioning patients to a new SPR NICU using the Canadian Interprofessional Health Collaborative (CIHC) National Interprofessional Competency Framework. This framework has been used successfully in aiding students in health professions to better understand the components of IPC and in designing a workshop to create IPC simulations [11,12].

The current study aimed to (1) identify and mitigate LSTs before transitioning patients to the new SPR NICU; (2) demonstrate the efficacy of IPC and ISS in identifying LSTs, and improving system readiness and staff preparedness before transitioning to a new HCE; and (3) share lessons learned to improve future transition in HCE.

## Materials and methods

Study setting

This was a single-center prospective simulation-based interprofessional intervention study conducted over approximately one year at Sainte-Justine's University Hospital (Montreal, Canada) in 2017, a women and children’s university hospital with a high-risk delivery service of 3500 births per year and a 65-bed level-3 NICU. A new building, including a SPR NICU, and a labor and delivery ward was built. The study was approved by the center's Institutional Review Board.

Participants

Neonatal HPs, trainees, and laboratory and radiology technologists were invited to participate in the study. On occasion, HPs from other specialties were also invited to participate. Parents of former NICU patients, members of the hospital parent advisory committee, and those whose children’s hospitalizations were remote were also invited to participate in the study. There were no exclusion criteria for neonatal HPs and parents.

Conceptual framework

Our interprofessional team used the CIHC National Interprofessional Competency Framework and its six domains to promote IPC in the development and implementation of simulation activities.

1. Interprofessional Communication: Ensured that all members were aware of the goals of the ISS and the transition plan, shared the common goal of identifying potential safety threats through the planning of simulation activities, and knew their roles/responsibilities in these processes. Team members used common technology-based tools and regular in-person meetings to achieve their common goals.

2. Patient-centered care: Parents of former hospitalized patients were included in the simulations and debriefings to provide the family perspective.

3. Role clarification: It was imperative that each team member understood their responsibilities related to planning and conducting simulation activities, identifying and addressing safety threats, debriefing, and follow-up tasks.

4. Team functioning: Team members encouraged collaboration among different healthcare disciplines to systematically assess the clinical environment for potential safety hazards. This was supported through the inclusion of a diversity of members as part of the educational team and facilitating discussion during regular effective team meetings.

5. Collaborative leadership: An interdisciplinary team of HPs was created for this project to plan, organize, and implement the simulation activities. The collaboration allowed the identification and mitigation of LSTs. A physician and a nurse educator led the team by sharing a common mission and common goals to achieve. Open constructive discussions were welcome to help the team move forward.

6. Interprofessional conflict resolution: The elements of the framework put in place by our working team enabled us to have the skills to address any disagreements or differing perspectives raised during the simulation process, ensuring that all safety concerns were identified and effectively addressed before the NICU relocation.

Simulation activities

Three different types of simulation activities were conducted.

Low-Fidelity Mock-Ups

Life-size mock-ups of a standard NICU patient room, stabilization room, and birthing room neonatal resuscitation area were created to assess and configure these areas before the completion of construction [13,14]. Twenty neonatal and obstetrics HPs (including medical directors, resuscitation committee leads, nurse and respiratory therapy educators), unit managers, an orderly, clerk, and cleaning personnel interacted with and manipulated the mocked-up environment that included articulated arms, cardiorespiratory monitors, compressed gas and electrical outlet locations, and patient beds, to determine optimal locations for equipment installation.

Tabletop Simulations

Using architectural plans and findings from the mock-ups, the same group of HPs conducted tabletop simulations to develop new workflows and assess the integration of existing and new workflows and processes in the new environment.

In Situ Simulations

Building on the findings of the mock-ups and table-top simulations, 12 HPs (physician, registered nurse, neonatal nurse practitioner, respiratory therapist, and pharmacist) with experience in simulation-based education created the ISS. Two simulation technicians assisted with planning and operationalizing the ISS. The simulation team garnered institutional leadership buy-in to secure access to the new NICU before post-construction finalization, as well as equipment and human resources to complete testing of the new NICU and with LST mitigation. The simulation team gained first access to the new unit 10 weeks before the transition.

Scenario development

Based on previous studies that used ISS to assess new NICU HCEs, and clinical priorities identified by the interprofessional simulation team and NICU leadership, we identified seven overarching objectives for the simulations (Appendices Table 4)[1,8]. Through an iterative process, the simulation team generated a list of 21 processes related to vital clinical activities to be tested during the ISS. Through close collaboration, the interprofessional team identified key procedures and workflows to be tested. Nine unique scenarios were created to include the spectrum of interprofessional clinical activities and associated workflow. Each scenario contained specific learning objectives and included routine clinical care and 2-3 specific activities (total of 21) such as resuscitation at birth, acute clinical deterioration, and in-hospital or out-of-hospital transport (see Appendices). Non-neonatal HPs, such as anesthesiologists, surgeons, interventional radiologists, obstetric teams, and paramedics were invited to participate in the scenarios, as appropriate.

Preparing the Environment

To mimic the completed clinical environment, 1/5 of the new NICU was stocked with authentic equipment and supplies and low- and high-technology mannequins. Vital sign simulators were connected to patient monitors. The communication systems were already in place and functional.

Conducting the ISS

**Figure 1:**
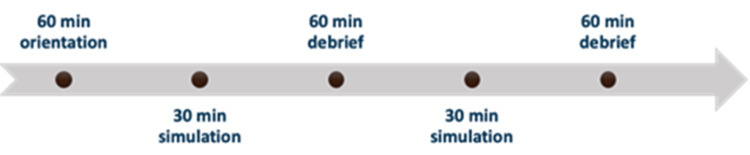
Timeline of each half-day (four hours in total) of the immersive in situ simulation program

Following a one-hour orientation to the new environment and technologies, a pre-briefing was conducted to: outline the importance of psychological safety, review mannequins and other simulation equipment, discuss the role of the simulation team and expectations of the participants, introduce the structure and goals of debriefing, and emphasize the goal of the simulations. NICU HPs and parents then participated in two 30-minute ISS during which the nine scenarios were run concurrently (Figure 1). A member of the simulation team was assigned to each scenario to facilitate and guide participants, as needed, (e.g. locating equipment, providing clinical cues not visible on the mannequin) and to record safety threats and concerns identified by the simulation team or simulation participants.

Parents were scripted in the role of parents to test processes, such as calling in for clinical updates, providing kangaroo care, pumping milk/breastfeeding, etc.

Post-Simulation Debriefing

**Figure 2:**
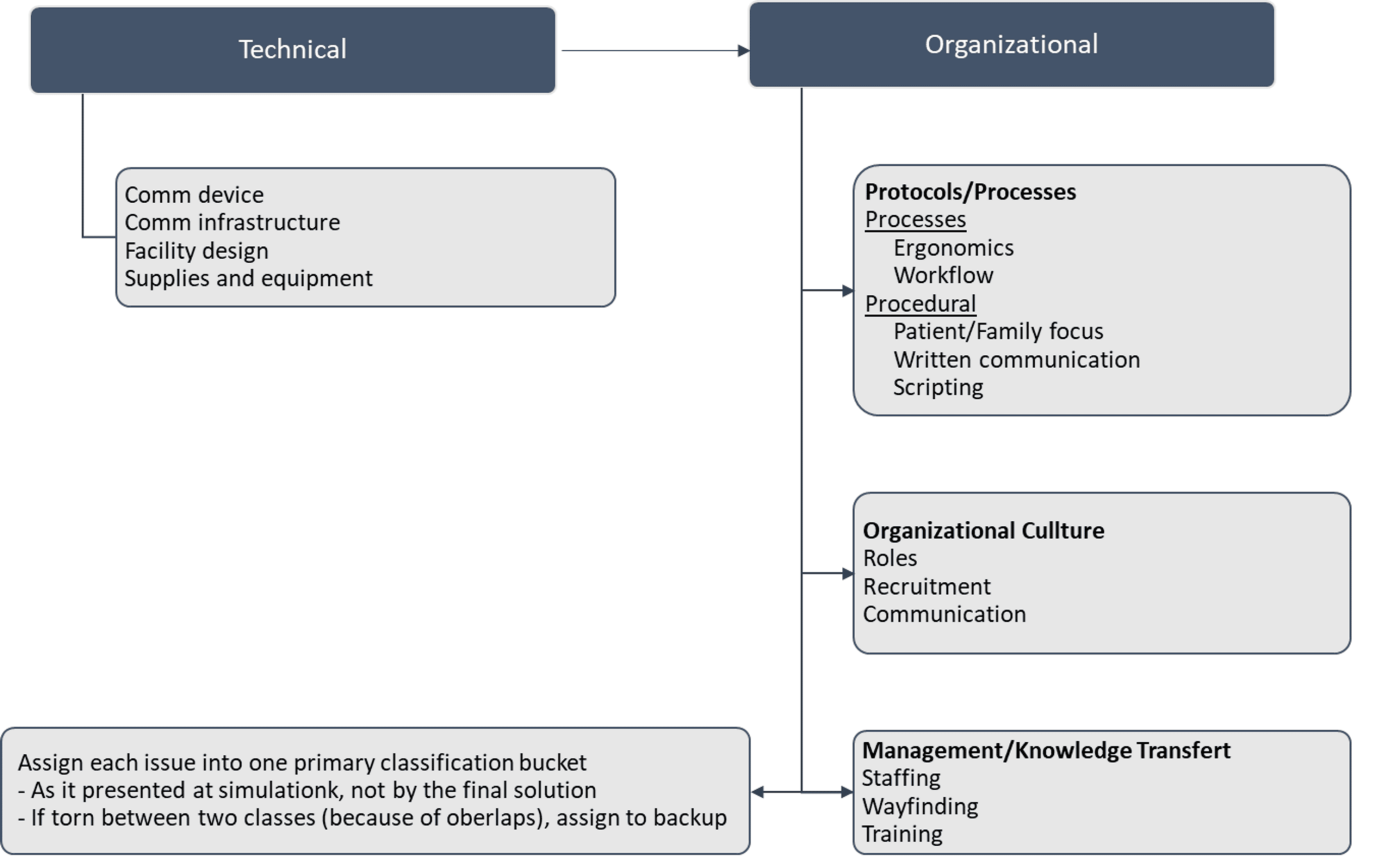
Latent safety threats classification system

Each ISS session was followed by a 60-minute debriefing, conducted by the project lead (Author-AM), an expert debriefer, using the PEARLS healthcare debriefing tool [15]. Should any residual issues in the scenario were not addressed in the debriefing, the simulation team members would verbalize them. The person in charge of the debriefing facilitated discussion to collate a detailed description of each issue and identify the existing or potential impact on professional teams or patients. The issues were then individually summarized as LSTs. Participants were also encouraged to suggest and discuss potential solutions. During the debriefing six of the interprofessional simulation team members documented the identified LSTs and potential solutions on a large paper clipboard. LSTs were categorized into themes, based on an LST classification system developed previously by two of the authors (Author-BR and Author-JB; Figure 2); organizational, building, ergonomics, communication, technical, and other issues. Documented issues were then transcribed electronically by the project leader.

Data collection

Debriefing Transcripts

Author AM and Author ALJ compared the content of the transcripts to the paper clipboard notes from the debriefings identifying missing findings and errors. Transcripts were then reviewed to identify redundancy and discrepancies. Transcript data was then separated into the final list of identified LSTs, possible solutions, and mitigation strategies.

Surveys

HPs were surveyed three times, using the same survey, during the transition: at baseline, post-ISS, and post-transition to the new HCE. The intermediary surveys documented the progression of systems readiness and staff preparedness and established internal consistency for each respondent. The survey included 71 questions that addressed 11 major themes and staff bias modulators. The latter included measures on emotional exhaustion, personal accomplishment, control over practice, handling conflict, staff relationships, and internal work motivation, as adapted from the Professional Practices Environment, MBI Human Services Survey, Family Centered Care Survey, and Expanded Nursing Stress Scales [16,17]. The language was adjusted to improve discernment of systems readiness from staff preparedness. Poorly worded questions with low Cronbach's alpha or factor analysis loadings were removed. An expert panel independently scored the survey to ensure that the classification of each question was primarily systems readiness or staff preparedness. Systems readiness and staff preparedness on 24 clinical processes were rated on a 5-point Likert scale (Appendices, Table 5).

Data analysis

Debriefing Transcripts

Author-AM and Author-ALJ reviewed the debriefing transcripts to ensure shared understanding and to reword statements for better coherence. Using qualitative methods, LSTs were categorized. A third researcher resolved discrepancies.

Surveys

Survey responses were analyzed using dependent samples t-tests and repeated measures ANOVAs (SPSS V25, Chicago, IL, USA).

Strategies for mitigating LSTs

After classification, the LSTs were distributed to the appropriate multidisciplinary working committees. For example, LSTs related to communication systems were sent to the institutional committees whereas LSTs related to the unit’s organizational processes went to the dedicated unit team. These committees, in place at the beginning of the transition project, were charged with reviewing processes and adapting workflows considering the transition. Correction of LSTs was requested within two to four weeks.

Simulation-based re-testing

To re-test the NICU environment and the efficacy of LST mitigation strategies additional ISS were conducted six and four weeks before transitioning to the new environment. The last four weeks before the transition were needed to plan the transition and train the remaining HPs to the new unit.

## Results

Six four-hour simulation sessions were conducted 10, six, and four weeks before transition. One hundred eight HPs (33%) including 52 (49%) nurses, 14 (13%) respiratory therapists, 10 (9%) physicians, 11 (10%) trainees, three (3%) nurse practitioners, seven (7%) orderlies, eight (8%) clerks, and one (1%) radiology technician, as well as 24 parents participated in the ISS. Eighty-nine LSTs were identified, and categorized into eight themes: work organization, orientation and parent wayfinding, communication devices/systems, nursing and resuscitation equipment, ergonomics, parent comfort, work processes, and interdepartmental interactions (Table 1). The majority (n=68; 76%) of the LSTs were resolved before the transition.

**Table 1:** Latent safety threats identified during immersive in situ simulation LSTs: latent safety threats; NICU: neonatal intensive care unit

Themes	N=89	Examples
Technical, n (%)		
Communication device	1 (1)	Portable devices did not receive alarms
Communication infrastructure	5 (6)	In patient rooms, it is not clear that the emergency codes were properly activated after the button was pressed
Facility design	8 (9)	Opened birthing room doors block access to the infant stabilization room
Supplies and equipment	8 (9)	The code cart is too remote from patient rooms
Organizational: protocols/processes		
Ergonomics	5 (6)	Wall air connections are far from the ventilators, resulting in hoses posing trip hazards
Workflows	7 (8)	The process of code blue (adult arrest in a NICU unit) needs to be better defined
Patient/family focus	0 (0)	No LSTs identified in this category
Written communication	4 (4)	The written message on hall screens when emergency buttons are activated is too small to read
Scripting	0 (0)	No LSTs identified in this category
Organizational: organizational culture		
Roles	6 (7)	During an emergency, establish which respiratory therapist will respond
Recruitment	19 (20)	Communication between team members (doctors, nurses) during codes to ensure that there is a response team
Communication	26 (29)	Intercom messages when the “nurse” and “respiratory therapist” help buttons are activated are similar and cause confusion as to who is requested
Organizational: management/knowledge transfer		
Staffing	1 (1)	There is a need for an extra resource nurse when a baby deteriorates in the next room
Wayfinding	0 (0)	No LSTs identified in this category
Training	0 (0)	No LSTs identified in this category

The survey response rate was 31%, 16%, and 7% for baseline, post-simulations, and post-transition, respectively (Table 2).

**Table 2:** Survey results at baseline, after simulations, and after relocation to single-family room Survey questions used a 5-point Likert scale and the results presented are average responses

	Baseline (n=93)	Post-simulations (n=48)	Post-relocation (n=21)	P-value (baseline vs. post-simulations)	P-value (baseline vs. post-relocation)
System readiness	1.3	3.5	3.9	<0.001	0.02
Staff preparedness	1.4	3.3	3.9	0.006	0.03

System readiness

HPs reported that system readiness increased post-simulations (p<0.001) and post-transition (p=0.02). Parents felt a lack of intimacy during kangaroo care and milk pumping due to glass doors, and hence opaque film was added to the lower two-thirds of the doors to create privacy. As it was difficult to read the location of codes on the corridor screens because of very small writing, the clerk was asked to call out the code on the overhead system.

Staff preparedness

HPs also reported improvement post-simulations (p=0.006) and post-transition (p=0.03). In the new unit, emergency drips would be prepared at the patient’s bedside as all necessary equipment was available in the resuscitation cart. HPs did not need to run to the supply room to fetch the equipment. In addition, the code system calling different HPs for different types of emergencies was confusing when the old system was transposed to the new unit. This was modified to clearly identify a different button for each emergency.

## Discussion

The aims of our prospective simulation-based study were as follows: identify and mitigate LSTs before transitioning patients to the new SPR NICU, demonstrate the importance and efficacy of IPC in conducting ISS before transitioning patients to a new HCE, and share lessons learned that can be generalized to IPC for ISS used for testing new HCEs.

Identification and mitigation of LSTs

Our IPC team identified 89 LSTs and allowed for the majority (n=68; 76%) to be resolved before transition. We described, supported by examples, improved system readiness and staff preparedness as an expected consequence of the quality improvement process. We hypothesize that our ISS prepared HPs to become familiar with the new HCE and increased their communication [18]. ISS is well recognized as an effective strategy to identify LSTs in various clinical settings [19-22]. It has also been used before transitioning to new HCEs, but the literature describing this transition in ICUs, particularly in the pediatric and neonatal setting, is scarce [1,23]. Our study adds to the current literature by describing the benefits of ISS in identifying LSTs before transitioning to a new HCE. Our study is the first to be performed in a large Canadian university hospital-based NICU. In addition, we also involved prior NICU parents in the ISS. This novel approach helped identify LSTs and generated recommendations that took the family’s perspective into account.

Other approaches have been used to plan the transition to a new HCE. Magdinsky et al. reported using preparation strategies that did not include simulation to plan the transition to a new SPR adult ICU [24]. While clinicians felt ready for the transition, challenges related to teamwork and patient care persisted after the transition period. We believe that including ISS in preparation for a transition helps alleviate those challenges. Williams et al. further support the financial and safety benefits of simulation-based testing [25]. They found that simulation-based clinical systems testing during transitions in pediatric trauma centers led to improved safety outcomes and cost savings by identifying and mitigating risks early. This aligns with our findings that simulation can significantly enhance system readiness and staff preparedness. In addition, Kaba and Barnes established that using ISS in these settings contributes to the development of a culture that shows openness and acceptance of simulation-based training [26]. In our unit, ISS has since been successfully implemented and we are currently studying its ongoing impact on quality improvement through teamwork and non-technical skills of HPs.

Importance and efficacy of IPC in conducting ISS

Our study not only outlines that simulation activities are crucial for transitioning to a new clinical environment but also emphasizes the importance of the CIHC National Interprofessional Competency Framework in guiding these efforts. Incorporating various simulation modalities - low-fidelity mock-ups, tabletop simulations, and immersive simulation scenarios - enabled engagement across multiple disciplines and HPs with diverse motivators or barriers. This experiential education strategy, rooted in adult learning theory, facilitated the identification of LSTs and promoted patient safety. Moreover, involving prior NICU parents brought credibility and humanism to the process, ensuring that the patient's perspective was valued and respected, which is vital for enhancing the NICU parenting experience [27]. While no objective evidence directly measures its impact, a parallel can be drawn with beneficial parental involvement during cardiopulmonary resuscitation, or even with the benefits of peer helper programs [28,29].

Other frameworks focussing on IPC have been published recently. The Team FIRST framework, developed by the Agency for Healthcare Research and Quality (AHRQ), complements the CIHC framework by offering specific strategies for enhancing teamwork, communication, and patient safety in healthcare settings [30]. While the latter outlines competencies for IPC, the former provides actionable guidance on fostering a culture of safety, promoting mutual respect among team members, and improving communication skills to enhance patient outcomes. By integrating the principles of Team FIRST with the competencies outlined in the CIHC framework, interprofessional teams working on educational or quality improvement initiatives can gain insights into practical tools and strategies for building effective teams.

Our findings are consistent with those of Sarwal et al. (2024) in the adult ICU and Dadiz et al. (2023) in the NICU, which demonstrated the efficacy of in situ simulations in identifying latent safety threats before transitioning to new HCEs [31,32]. Similarly, our study within the neonatal population revealed that in situ simulations are instrumental in detecting and mitigating safety threats, thereby enhancing patient safety and care efficiency. These results underscore the critical importance of such simulations before any transition to new healthcare facilities. Notably, our study distinguishes itself by incorporating parent partners and emphasizing the interdisciplinary collaboration essential for the successful execution of in situ simulations.

Lessons learned

We believe that ISS, while time-consuming, benefits clinicians by allowing them to design and appropriate new HCE better. Furthermore, interprofessional ISS involving HPs, and patients and families, should become an essential element in the process of planning a transition. ISS is time-consuming and requires a certain number of human resources in terms of education and simulation expertise in addition to simulation equipment and healthcare supplies. However, in lower-resource hospitals, it is beneficial to conduct ISS before transition with low-fidelity simulation equipment and the use of expired healthcare supplies to limit the costs. For future interprofessional teams willing to use simulation to test a new HCE before a transition, we would like to share certain key recommendations to ensure the success of the interprofessional quality improvement initiative (Table 3).

**Table 3:** Key recommendations to ensure success of the interprofessionnal quality improvement initiatives HPs: healthcare professionals; LSTs: latent safety threats

Key principles	Comments
Build a team of dedicated interprofessional HPs with SBE expertise	
Keep in mind the principles of SBE	Identify clear objectives for each component of the simulations
	Recruit an expert in debriefing as the approach to identifying LSTs is different than regular educational simulations and debriefings tend to include a large group of participants
	Build scenarios to represent reality as closely as possible
	Ensure participant’s psychological safety during the simulations
Ensure collaborative leadership to facilitate the operationalization of the simulations and mitigation of LSTs	
Plan clear workflows with quality improvement committees that are responsible for mitigating the LSTs	
Once LSTs have been mitigated, plan for re-testing the environment with simulation	
Include all HPs involved in patient care and teams outside the unit to gain further perspectives	
Patient and/or parent involvement is essential to highlight patient-centered issues and specific needs	

This study has a few limitations, primarily its single-center design. Second, the survey response rate decreased by half after each iteration, which could have caused a selection bias, thereby overestimating the results. The low response rate can be explained by the extra workload added by the transition, a particularly busy period for the NICU team.

## Conclusions

This prospective study highlights the importance of interprofessional collaboration in conducting ISS before transitioning to a new HCE. Coordinating large-scale simulations is a worthwhile investment of time and resources, as it helps identify LSTs, optimize system readiness, and promote patient safety. Other hospitals can learn from this study by recognizing the value of ISS in proactively addressing potential risks during transitions. ISS should be incorporated into standard transition protocols to ensure comprehensive preparation and enhance patient safety. We hope that the lessons shared in this study will assist future interprofessional teams in planning and executing transitions to other HCEs. We call on healthcare institutions to adopt ISS as a critical component of their transition strategies to safeguard patient safety and improve outcomes.

## Appendices

**Table 4:** General objectives, clinical activities, and simulated patients used for in situ simulations CDH: congenital diaphragmatic hernia; DOL: day of life; HFNC: high-flow nasal cannula; HIE: hypoxic-ischemic encephalopathy; iNO: inspired nitric oxide; LFNC: low-flow nasal cannula; NICU: neonatal intensive care unit; PAH: pulmonary arterial hypertension; RA: room air; UAC: umbilical arterial catheter; UVC: umbilical venous catheter

Simulated patients (9)	Clinical activities (23)	Objectives (7)
29 weeks, DOL 5, CPAP 21%, gavages in progress, total parental nutrition on a picc line (Mathis)	Emergency code in the labor and delivery unit including the stabilization rooms. Admission of a newborn to the NICU from the labor and delivery ward	Patient movements (internal and external). Internal communication within the unit and external communication within the hospital and outside
The term, DOL 3 congenital heart defect on prostaglandins. Drinking small amounts of milk (Adam)	Transfer of a patient from the current mother-child units to the NICU. Code Pink on the current mother-child unit. Central line insertion in the NICU	Compliance with infection control policies (hand hygiene process at various entry points on the unit and in the rooms). Patient movements (internal and external). Internal communication within the unit and external communication within the hospital and outside. Appropriation of the premises, orientation in the space, repairs, and signage. Management of equipment of healthcare material
Ex 24 weeks now 34 weeks, HFNC 2LPM, feeding and learning to feed (Camille)		Appropriation of the premises, orientation in the space, repairs, and signage
Polymalformative syndrome, feeding, LFNC, under investigation (Nathalia)	Patient movements to medical imaging. Code blue of a NICU baby in medical imaging	Patient movements (internal and external). Internal communication within the unit and external communication within the hospital and outside. Appropriation of the premises, orientation in the space, repairs, and signage
28 weeks 2 weeks old, full feeds, RA (Ryan)	Transferring a patient from intermediate care to intensive care and vice versa in the NICU. Code Pink (newborn emergency) in the NICU, both in the intensive and the intermediate care. Deterioration of a patient in the NICU (call for respiratory therapist, call for MD, etc.). Deterioration of a baby in intermediate care and transferred urgently to intensive care	Patient movements (internal and external). Patient monitoring (alarm management and family support). Internal communication within the unit and external communication within the hospital and outside. Management of equipment of healthcare material. Movement through the unit of healthcare providers, consultants, relatives, and visitors
24 weeks DOL 2, UAC, and UVC in place, intubated, and tube fed (Naya)	Moving a patient from labor and delivery or operating room to the NICU. Emergency code in the labor and delivery unit including the stabilization rooms. Deterioration of a patient in the NICU (call for respiratory therapist, call for MD, etc.). Resuscitation in the stabilization room in the labor and delivery ward. Admission of a newborn to the NICU from the labor and delivery ward. Birth of a NICU patient in the labor and delivery ward	Patient movements (internal and external). Internal communication within the unit and external communication within the hospital and outside
36 weeks, pre- or post-op CDH, PAH on HFOV, iNO, NPO gomco, vasopressors, etc. (Justin)	Moving patients to the operating room. Moving a patient from labor and delivery or operating room to the NICU. Emergency code in the labor and delivery unit including the stabilization rooms. Resuscitation in the stabilization room in the labor and delivery ward. Admission of a newborn to the NICU from the labor and delivery ward. Birth of a NICU patient in the labor and delivery ward. Routine care of a complex case (HFOV, iNO, vasopressors, multiple medications, IV solutions, etc.). Admission of a complex baby (consultants, multiple technical platforms)	Patient movements (internal and external). Patient monitoring (alarm management and family support). Internal communication within the unit and external communication within the hospital and outside. Appropriation of the premises, orientation in the space, repairs, and signage. Management of equipment of healthcare material. Movement through the unit of healthcare providers, consultants, relatives, and visitors
34 weeks oesophageal atresia, replogle, anastomotic leak, and mediastinal infection (Beatrice)	Code Blue in the NICU (parent, visitor, or staff). Elective and emergency intubation in the NICU	Compliance with infection control policies (hand hygiene process at various entry points on the unit and in the rooms). Patient monitoring (alarm management and family support). Internal communication within the unit and external communication within the hospital and outside. Management of equipment of healthcare material
41 and 6 weeks cooling for HIE (William)	Neonatal transport simulation: emergency transport team departure. Return from transport and admission of a patient to a NICU room. Admission of a complex baby (consultants, multiple technical platforms). Central line insertion in the NICU	Compliance with infection control policies (hand hygiene process at various entry points on the unit and in the rooms). Patient movements (internal and external). Internal communication within the unit and external communication within the hospital and outside. Appropriation of the premises, orientation in the space, repairs, and signage. Management of equipment of healthcare material. Movement through the unit of healthcare providers, consultants, relatives, and visitors

**Table 5:** Questionnaire used to measure system readiness and staff preparedness Select ‘’N/A’’ if this process is not part of your job; select ‘’I don’t know’’ if it is typically part of your job, but you are uncertain about its state

These questions are about system readiness and staff preparedness. Think about your daily routine as it is now. How well have these processes been adapted for the new environment?								
Rate system readiness of the new NICU								
	N/A	I don’t know	Strongly disagree	Disagree	Inclined to disagree	Inclined to agree	Agree	Strongly agree
Equipment access (e.g., glucometer, warmer, pump, ventilator)								
Restocking bedside supplies								
Recruiting a team with the appropriate skills for a code situation								
Coordinated movement of staff and equipment at the bedside during a code blue resuscitation								
Communication systems for requesting urgent medical treatment								
Procedures for using individual communication devices								
Signage inside and outside patient rooms								
Critical laboratory results consistently reported to the correct practitioner								
Bedside monitor alarms get to the responsible nurse								
Nursing can readily identify the patient’s daily medical provider								
Protocols are clearly defined for verbal orders								
Getting drip medications urgently to the correct bedside								
Workflow for my specialty (e.g., patient assignment, report, documentation)								
Patient coverage when RN is unavailable (e.g., during lunchtime or procedures)								
Admitting a sick baby from the LDR								
Protocol for managing breastmilk (storage, mixing, fortifiers, distribution)								
The structure of team rounds in each area (e.g., sequence, participants)								
Medical model/team coverage (e.g., resident/NNP, night/day)								
Scripted responses for managing inappropriate parental behaviors								
Patient care privacy								
Mechanism for providing suggestions/improvements								
Please comment on other important processes								
Rate staff preparedness for the same processes: I feel prepared to perform this process in the new NICU								
Equipment access (e.g., glucometer, warmer, pump, ventilator)								
Restocking bedside supplies								
Recruiting a team with the appropriate skills for a code situation								
Coordinated movement of staff and equipment at the bedside during a code blue resuscitation								
Communication systems for requesting urgent medical treatment								
Procedures for using individual communication devices								
Signage inside and outside patient rooms								
Critical laboratory results consistently reported to the correct practitioner								
Bedside monitor alarms get to the responsible nurse								
Nursing can readily identify the patient’s daily medical provider								
Protocols are clearly defined for verbal orders								
Getting drip medications urgently to the correct bedside								
Workflow for my specialty (e.g., patient assignment, report, documentation)								
Patient coverage when RN is unavailable (e.g., during lunch time or procedures)								
Admitting a sick baby from the LDR								
Protocol for managing breastmilk (storage, mixing, fortifiers, distribution)								
The structure of team rounds in each area (e.g., sequence, participants)								
Medical model/team coverage (e.g., resident/NNP, night/day)								
Scripted responses for managing inappropriate parental behaviors								
Patient care privacy								
Mechanism for providing suggestions/improvements								
Please comment on other important processes								
